# Impact of the inversion time on regional brain perfusion estimation with clinical arterial spin labeling protocols

**DOI:** 10.1007/s10334-021-00964-7

**Published:** 2021-10-13

**Authors:** Francesco Sanvito, Fulvia Palesi, Elisa Rognone, Leonardo Barzaghi, Ludovica Pasca, Giancarlo Germani, Valentina De Giorgis, Renato Borgatti, Claudia A. M. Gandini Wheeler-Kingshott, Anna Pichiecchio

**Affiliations:** 1 Unit of Radiology, Department of Clinical, Surgical, Diagnostic, and Pediatric Sciences, University of Pavia, Viale Camillo Golgi, 19, 27100 Pavia, Italy; 2 Department of Brain and Behavioral Sciences, University of Pavia, Via Forlanini 6, 27100 Pavia, Italy; 3 Brain Connectivity Center Research Department, IRCCS Mondino Foundation, Via Mondino 2, 27100 Pavia, Italy; 4 Advanced Imaging and Radiomics, Department of Neuroradiology, IRCCS Mondino Foundation, Via Mondino 2, 27100 Pavia, Italy; 5 Department of Mathematics, University of Pavia, Via Adolfo Ferrata 5, 27100 Pavia, Italy; 6 Department of Child Neurology and Psychiatry, IRCCS Mondino Foundation, Via Mondino 2, 27100 Pavia, Italy; 7 NMR Research Unit, Department of Neuroinflammation, Queen Square MS Centre, UCL Queen Square Institute of Neurology, Faculty of Brain Sciences, University College London, Gower Street, WC1E 6BT London, England, UK

**Keywords:** Arterial spin labeling, Inversion time, Post-labeling delay, Cerebral blood flow, Brain perfusion

## Abstract

**Objective:**

Evaluating the impact of the Inversion Time (TI) on regional perfusion estimation in a pediatric cohort using Arterial Spin Labeling (ASL).

**Materials and methods:**

Pulsed ASL (PASL) was acquired at 3 T both at TI 1500 ms and 2020 ms from twelve MRI-negative patients (age range 9–17 years). A volume of interest (VOIs) and a voxel-wise approach were employed to evaluate subject-specific TI-dependent Cerebral Blood Flow (CBF) differences, and grey matter CBF *Z*-score differences. A visual evaluation was also performed.

**Results:**

CBF was higher for TI 1500 ms in the proximal territories of the arteries (PTAs) (e.g. insular cortex and basal ganglia ﻿—﻿ *P* < 0.01 and *P* < 0.05 from the VOI analysis, respectively), and for TI 2020 ms in the distal territories of the arteries (DTAs), including the watershed areas (e.g. posterior parietal and occipital cortex — *P* < 0.001 and *P* < 0.01 from the VOI analysis, respectively). Similar differences were also evident when analyzing patient-specific CBF *Z*-scores and at a visual inspection.

**Conclusions:**

TI influences ASL perfusion estimates with a region-dependent effect. The presence of intraluminal arterial signal in PTAs and the longer arterial transit time in the DTAs (including watershed areas) may account for the TI-dependent differences. Watershed areas exhibiting a lower perfusion signal at short TIs (~ 1500 ms) should not be misinterpreted as focal hypoperfused areas.

**Supplementary Information:**

The online version contains supplementary material available at 10.1007/s10334-021-00964-7.

## Introduction

Arterial spin labeling (ASL) is a non-invasive magnetic resonance (MR) technique that enables the in-vivo evaluation of regional brain perfusion by employing the magnetically-labeled inflowing blood as an endogenous tracer [1, 2]. Blood labeling is performed by inverting or saturating the magnetization of water molecules in the feeding arteries. The brain is imaged after a certain time interval in order to allow the labeled protons to flow towards the brain tissue [3]. Such time interval (from labeling to image acquisition) is usually called post-labeling delay (PLD) in pseudo-continuous ASL (PCASL) sequences and inversion time (TI) in pulsed ASL (PASL) sequences, whereas ‘arterial transit time’ (ATT) refers to the time necessary for the labeled blood water molecules to reach brain tissues [4]. The choice of PLD/TI is a compromise between the ability of measuring the effective perfusion signal only in tissues (rather than in blood vessels) and the possibility to obtain a higher signal-to-noise ratio (SNR). Indeed, a high PLD/TI value, i.e. greater than a typical ATT, means avoiding signal from intraluminal labeled water molecules that are still in the arteries, but also results in a lower SNR, as the labeling decay is directly proportional to the value of PLD/TI [4, 5]. It is worth mentioning that the labeling decay takes place in the capillary bed for most of the labeled water molecules [6], and that the residual labeled water molecules are free to diffuse into the interstitial space [3]. Therefore, extending PLD/TI does not result in sampling signal from venous vessels.

The white paper by Alsop and colleagues [5] provided some guidelines regarding these parameters. Following their recommendations, in both PCASL and PASL, the PLD/TI should be set as follows: 2000 ms for newborns, 1500 ms for children, 1800 ms for healthy adults (< 70 years old), and 2000 ms for adult patients and healthy subjects beyond 70 years of age. However, multiple ASL studies employed a PLD/TI of ~ 1500 ms for adult patients [7, 8], while others employed a PLD/TI of 1025 ms on pediatric subjects and newborns for the assessment of epileptic seizures [9, 10]. Finally, values of PLD/TI are not reported in some studies applying ASL to pediatric patients with epilepsy [11, 12].

Overall, the importance of choosing a correct PLD/TI is recognized but is not fully characterized, and evidence regarding the impact of PLD/TI on cerebral blood flow (CBF) estimation is scarce in the pediatric population. The main aim of this study was to evaluate the TI-dependent CBF variation in a cohort of pediatric patients (most of whom diagnosed with epilepsy) by performing PASL with two different TIs on each subject. The TI recommended in the white paper by Alsop and colleagues for the pediatric population (1500 ms) was compared to a longer TI (2020 ms), hypothesizing that a longer TI would result in signal decay and CBF underestimation. A secondary goal was to assess whether CBF is globally affected by TI or there are region-dependent TI-dependent CBF variations. This possibility has relevant clinical implications, for example on the evaluation of seizure-induced perfusion alterations, given that the epileptic focus may be detected as an either hypo- or hyperperfused cortical area on ASL when compared to the rest of the cortex [13].

## Materials and methods

### Subjects

All the enrolled patients underwent MRI as part of their clinical work-up. The study was carried out in accordance with the Declaration of Helsinki and patients’ parents gave consent to use the datasets for research purposes. This study was approved by the local ethic committee.

Inclusion criteria were as follows: absence of structural abnormalities on conventional MRI, age comprised between 9 and 18 years, MRI-datasets surviving visual quality assessment (see “MRI protocol”).

We selected twelve patients meeting the inclusion criteria: 7 females and 5 males (13.7 ± 2.8 years; range 9.6–17.4). Nine patients underwent MRI for epileptic seizures, and their MRI datasets were also included in other studies carried out by our Institution, assessing the correspondence between ASL-findings and clinical/EEG-findings in epilepsy [14]. The remaining three patients underwent MRI for headache, a new-onset visual impairment, and psychiatric symptoms (anxiety and depression), respectively.

In order to assess the reproducibility of our perfusion estimates regardless of the TI change, we also enrolled 5 healthy adults for a scan–rescan analysis: 3 females and 2 males (28 ± 1.25 years; range 26–29).

### MRI protocol

All patients were imaged on a Siemens Skyra 3 T scanner (Siemens Healthineers, Erlangen, Germany) with a 32-channel head-coil. The protocol included the following sequences in a single MRI-session:

- Two 3D gradient-and-spin-echo (GRASE) PASL PICORE Q2TIPS sequences, with identical parameters (TR/TE = 3500/21.1 ms, 20 axial-slices, FOV = 240 × 240 mm, matrix size = 128 × 128, reconstructed-voxel size = 1.875 × 1.875x5.25 mm^3^, flip-angle = 130°, 8 label/control pairs, background suppression, EPI-factor = 31, turbo-factor = 12, segmentation-factor = 4) except for TI, which was set to 1500 ms (ASL1500) and 2020 ms (ASL2020), respectively.
- Three ASL control images without background-suppression and with the same parameters as PASL, except for TI = 1000/1990/4000 ms and for TR = 1270/2270/4270 ms, respectively.
- One 3DT1 MPRAGE (TR/TE = 1900/2.3 ms, FOV = 210 mm, 144 sagittal-slices, voxel = 1.1 × 1.1 × 1.1 mm^3^).
- All MRI sequences considered useful for clinical purposes.

The order of the sequences was as follows: MRI diagnostic protocol (including 3DT1), ASL1500, ASL control images, ASL2020. A quality check of the acquired datasets was performed in order to verify the absence of artifacts such as the ones due to motion [15], and that background suppression and phase encoding directions were correctly set.

Depending on patient collaboration, MRI was performed either with (*n* = 3) or without (*n* = 9) sedation using sevoflurane, which has been reported to not significantly affect ASL-measurements [16]. It is important to notice that, when the sedation was required, it was employed for the entire MRI session. Therefore, ASL1500 and ASL2020 were both acquired either with or without sedation, so that the sedation did not affect the assessment of TI-dependent differences.

For the scan–rescan analysis, the healthy adults underwent the same MRI protocol, with the exception of TI being set at 1800 ms for both PASL sequences (‘ScanA’ and ‘ScanB’), as recommended for their age in the white paper [5].

### CBF quantification

For each patient, DICOM-images were converted to NifTI-format, then the built-in commands of the FMRIB Software Library (FSL, University of Oxford, https://fsl.fmrib.ox.ac.uk/fsl/) were employed for motion correction of ASL images (‘mcflirt’), to linearly align M0 and ASL2020 images to the midvolume control image of the ASL1500 sequence (‘flirt’), to register the resulting aligned images to the 3DT1 using an affine transformation (‘flirt’), and to normalize the 3DT1 volume to the MNI152 template using a non-affine transformation (‘fnirt’). ASL1500 will be considered the native space of each patient.

The following steps were performed in native space for both ASL1500 and ASL2020. ASL labeled/control difference obtained through ‘asl_file’ command (FSL) generated the ASL perfusion-weighted map. The three M0 images were used in ‘asl_calib’ for calibration and to correct for the equilibrium magnetization of arterial blood. Then, the absolute CBF (i.e. CBF1500 and CBF2020) was quantified by applying the standard formula as seen in the white paper by Alsop et al. [5], with TI_1_ (bolus duration) set to 700 ms.

### Brain parcellation and regional CBF evaluation

The FSL ‘fast’ segmentation was applied to 3DT1 to obtain binary masks of grey matter, white matter, and cerebrospinal fluid. Grey and white matter masks were transformed in the native space by inverting the affine registration calculated in “CBF quantification”.

On the basis of the Harvard–Oxford Atlas (https://fsl.fmrib.ox.ac.uk/fsl/fslwiki/Atlases) [17], grey matter was parcellated in 7 volumes of interest (VOIs) for each hemisphere: frontal, parietal, temporal, occipital, and insular cortex, temporo-mesial structures (including amygdala and hippocampus), and basal ganglia (including caudate, putamen, internal pallidum, external pallidum). For the white matter, a VOI was obtained by selecting the supratentorial white matter from the structural segmentations available in FSL MNI-space. These VOIs were transformed to the ASL native space by inverting and concatenating (‘applywarp’) the affine and non-affine transformations calculated in “CBF quantification”. In order to minimize partial volume effects, for each patient, cortical VOIs were intersected with the ‘fast’ grey matter segmentation, and the white matter VOI was intersected with the ‘fast’ white matter segmentation.

For both CBF1500 and CBF2020, regional mean-CBF values were extracted for the whole cerebral cortex (voxels belonging to all cortical VOIs), the supratentorial white matter, basal ganglia, and each cortical VOI. For each cortical VOI, a *Z*-score (*Z*_VOI_) was also calculated as follows:

$$Z_{{{\text{VOI}}}} = \frac{{{\text{CBF}}_{{{\text{VOI}}}} - \overline{{{\text{CBF}}}}_{{{\text{cortex}}}} }}{\sigma },$$

where VOI refers to a specific cortical VOI, $\overline{{{\text{CBF}}}}_{{{\text{cortex}}}}$ indicates mean CBF of all voxels belonging to the cerebral cortex for each patient and *σ* is the corresponding standard deviation. *Z*-scores reflect the regional perfusion of a specific cortical VOI compared to the whole cerebral cortex. More in detail, *Z*_VOI_ corresponds to the number of standard deviations of cortical CBF (*σ*) that separate CBF_VOI_ from $\overline{{{\text{CBF}}}}_{{{\text{cortex}}}}$, with: *Z*_VOI_ ~ 0 when CBF_VOI_ ~ $\overline{{{\text{CBF}}}}_{{{\text{cortex}}}}$; *Z*_VOI_ > 0 when CBF_VOI_ > $\overline{{{\text{CBF}}}}_{{{\text{cortex}}}}$; *Z*_VOI_ < 0 when CBF_VOI_ < $\overline{{{\text{CBF}}}}_{{{\text{cortex}}}}$. This index is an easy-to-read metric reflecting whether the perfusion in a cortical region is higher or lower compared to the whole cortex and to what degree. Employing such score is useful because in the clinical setting ASL evaluation is mostly performed through a qualitative visual assessment of perfusion maps. During the qualitative assessment, neuroradiologists identify cortical areas exhibiting a higher or lower perfusion signal compared to the contralateral and/or adjacent areas. Therefore the relative perfusion of a cortical region compared to the rest of the cortex has a relevant clinical significance.

### Statistical analysis

Despite some previous studies attempted to provide reference perfusion metrics in pediatric subjects [16, 18], regional CBF in the pediatric population is strongly age-dependent and no reliable reference values for ‘normal’ ASL-derived CBF are available for comparison. Therefore, we evaluated TI-dependent differences only by comparing perfusion data within the same subject. All variables, i.e. regional CBF (cerebral cortex, white matter, basal ganglia, cortical VOIs) and *Z*-scores (cortical VOIs) values, were compared within each subject employing a paired Wilcoxon signed-rank test in order to evaluate the VOI-specific TI-dependent differences. The choice of a paired test prevented the analysis from being biased by CBF asymmetries due to the underlying condition (e.g. an epileptic focus). The reason for choosing a non-parametric test (Wilcoxon) was that not all variables passed a D’Agostino-Pearson normality test. Benjamini–Hochberg procedure was employed to adjust for the false discovery rate. We set the significant threshold at Wilcoxon *P* < 0.05 surviving Benjamini–Hochberg adjustment.

### Voxel-wise analyses of TI-dependent changes

In each patient, subtracting CBF1500 and CBF2020 maps from each other produced patient-specific CBF subtraction-maps depicting voxel-wise TI-dependent CBF differences. In order to provide an ‘across-subjects’ assessment of these TI-dependent differences, all patient-specific subtraction-maps were binarized, normalized to the MNI152 space using a nearest-neighbor interpolation, and added together.

For each patient, a TI-dependent voxel-wise *Z*-score map (i.e. *z*-1500 and *z*-2020) of grey matter CBF was calculated by including all voxels from the ‘fast’ grey matter mask. For each voxel belonging to the grey matter, the *Z*-score *(Z*_voxel_*)* was calculated as follows:

$$Z_{{{\text{voxel}}}} = \frac{{{\text{CBF}}_{{{\text{voxel}}}} - \overline{{{\text{CBF}}_{{{\text{grey}}}} }} }}{\sigma },$$

where voxel refers to a specific grey matter voxel, $\overline{{{\text{CBF}}_{{{\text{grey}}}} }}$ indicates mean CBF of the voxels belonging to the grey matter for each patient, and *σ* is the corresponding standard deviation. *Z*_voxel_ are the voxel-wise counterpart of the previously defined *Z*_VOI_, and they reflect the regional perfusion of a specific voxel compared to that of the other grey matter voxels. The mathematical relationships described among *Z*_VOI_, CBF_VOI_ and $\overline{{{\text{CBF}}}}_{{{\text{cortex}}}}$ also exist among *Z*_voxel_, CBF_voxel_ and $\overline{{{\text{CBF}}_{{{\text{grey}}}} }}$. *Z*_VOI_ and *Z*_voxel_ provide complementary information, the former being more robust for statistical analyses and the latter being characterized by a higher spatial resolution (since it also depicts the relative perfusion of voxels belonging to the same VOI). Voxel-wise computation of *Z*-scores has already been proposed in previous studies, including other ASL studies [19, 20], to obtain indexes representing voxel values relatively to other voxels.

*z*-1500 and *z*-2020 were subtracted from each other to obtain patient-specific *Z*-score subtraction-maps and across-subjects *Z*-score subtraction-maps were obtained as for the aforementioned CBF subtraction-maps.

### Scan–rescan analysis

The same pipeline described for pediatric patients was applied to healthy adults for the following steps: CBF quantification; brain parcellation and CBF extraction from VOIs; Wilcoxon signed-rank test (with Benjamini–Hochberg adjustment) to assess scan–rescan CBF differences in each VOI; generation of voxel-wise CBF subtraction-maps (subtracting ScanA and ScanB from each other) for an ‘across-subject’ assessment.

### Visual assessment of perfusion maps

As an additional analysis, in order to provide a clinical assessment of perfusion at different TIs and to highlight TI-dependent differences at single-subject level, a qualitative visual evaluation was performed. TI-dependent differences were qualitatively evaluated by uploading ASL-datasets on a clinical software product (Olea Sphere Software 3.0 ©) and computing qualitative perfusion-weighted maps for each TI. This clinical software product was chosen because it generates high-quality perfusion maps, and allows to constantly adjust window level and width during the visual evaluation, as well as the degree of overlay with morphological images. For each subject, the resulting TI 1500 qualitative perfusion-weighted map was visually compared to the resulting TI 2020 qualitative perfusion-weighted map. The comparison aimed at identifying which cerebral regions showed a TI-dependent difference in perfusion signal, and the regional perfusion compared to the adjacent regions at different TIs. The use of a clinical software product for this qualitative assessment was chosen in order to reproduce the standard work-flow for the evaluation of single-subject ASL datasets in the clinical setting.

Similarly, for each subject, quantitative CBF maps computed with FSL (i.e. CBF1500 and CBF2020) were visually compared, in order to assess whether the TI-dependent differences visualized with the clinical software product were also apparent in the quantitative maps.

### Impact of sedation and age on TI-dependent changes

As additional analyses, we assessed whether sedation and age had an impact on TI-dependent CBF changes. For both analyses, we subtracted subject-specific CBF2020 from CBF1500 values with a VOI-based approach, in order to obtain VOI-specific TI-dependent CBF-variations (CBF2020–CBF1500; [ml/min/100 g]).

To evaluate the impact of sedation, we compared such CBF-variations [ml/min/100 g] between sedated patients (*n* = 3) and age-matched (age range 9–16 years) awake patients (*n* = 5), using a Mann–Whitney *U* test (significant threshold: *P* < 0.05) and a Benjamini–Hochberg procedure to adjust for the false discovery rate.

To evaluate the impact of age, a Pearson correlation coefficient (rho) was calculated between CBF-variations [ml/min/100 g] and age [years], only in awake patients (*n* = 9). The absolute values of rho coefficients were interpreted as follows: < 0.2 negligible correlation, 0.2–0.4 weak correlation, 0.4–0.6 moderate correlation, 0.6–0.8 strong correlation, > 0.8 very strong correlation [21].

## Results

**Fig. 1:**
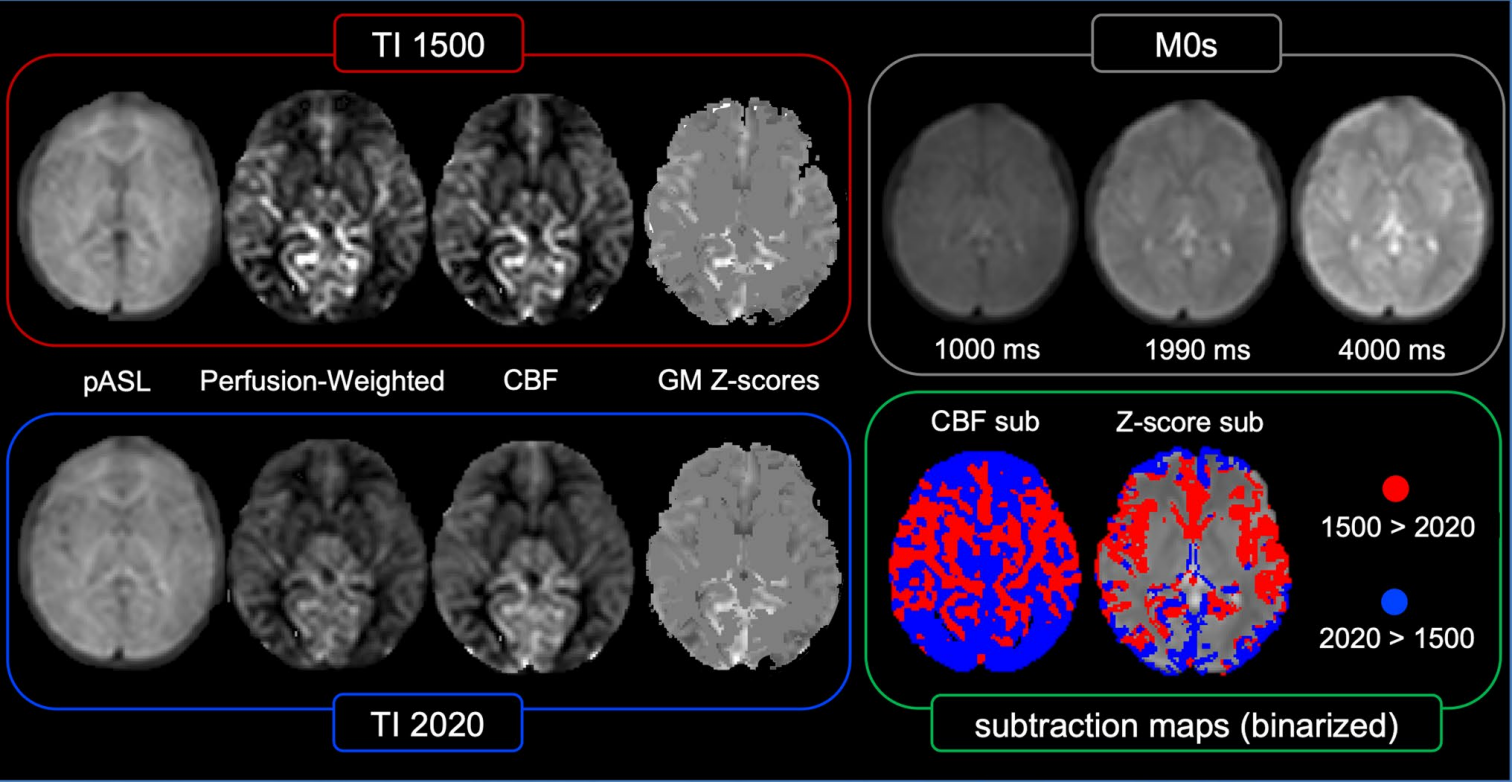
Representative datasets and ASL-derived maps in a single patient, randomly selected. Acquired images include PASL at TI 1500 ms (red square) and TI 2020 ms (blue square), as well as M0 control images (grey square). For each TI, PASL datasets were used to generate perfusion-weighted maps, CBF maps, and grey matter (GM) *Z*-score maps (i.e. z1500 and z2020). Finally, CBF and *Z*-score maps from each TI were subtracted and binarized (green square)

Single-subject representative datasets and maps are shown in Fig. 1.

### Differences in CBF

**Fig. 2:**
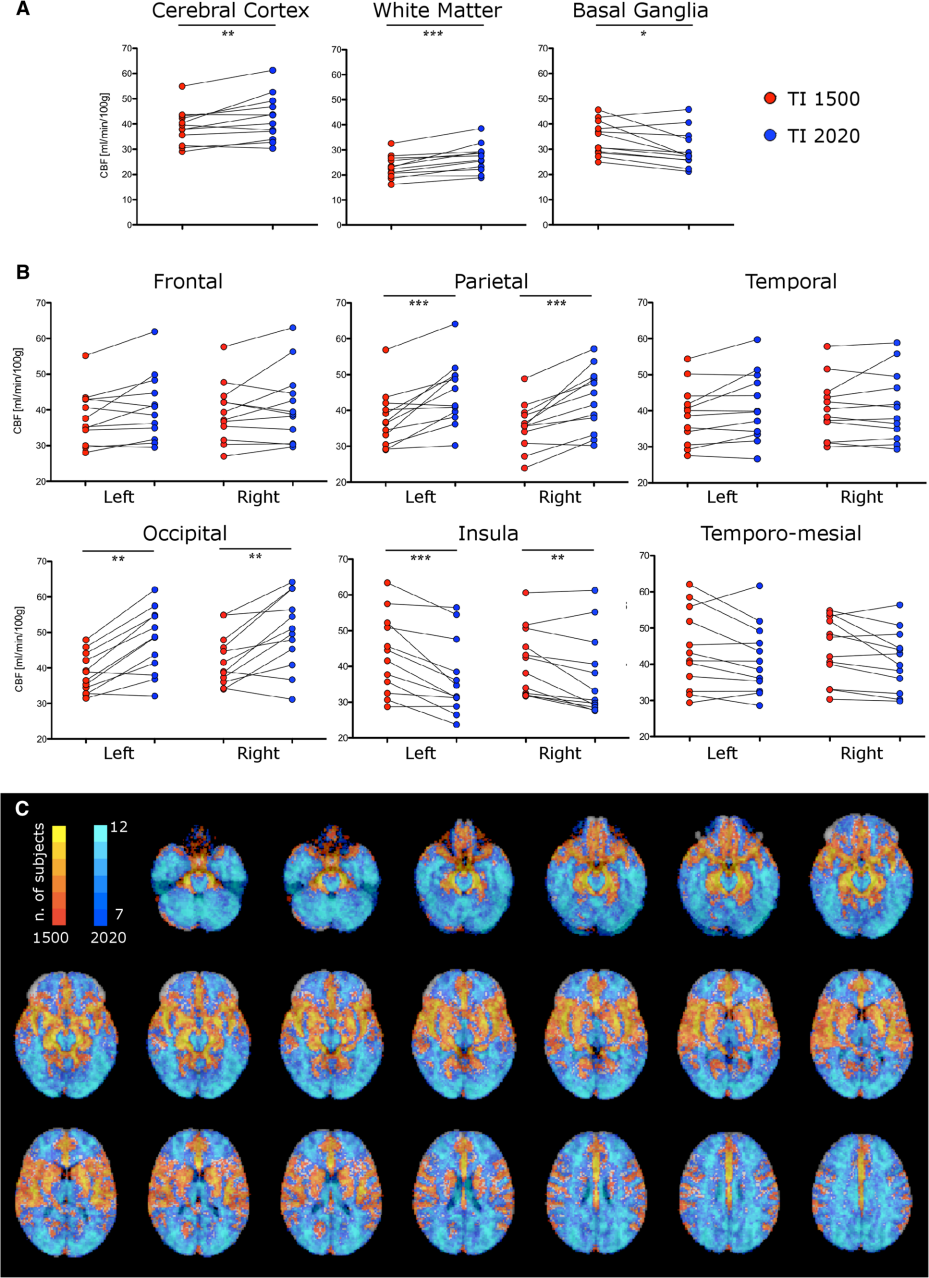
TI-dependent differences of CBF. Box plots show regional CBF differences (**A**, **B**); *p < 0.05, **p < 0.01, ***p < 0.001, – only Wilcoxon significant p-values surviving Benjamini–Hochberg adjustment are displayed. Voxel-wise CBF across-subjects subtraction-maps (**C**) illustrate voxels with higher CBF1500 (red) and higher CBF2020 (blue); color shade is proportional to the number of subjects

Figure 2 shows all TI-dependent differences of absolute CBF both in regional and voxel-wise fashion. The regional values of CBF are reported in Tab.1. Statistical comparisons of regional metrics showed that CBF1500 was significantly higher in the basal ganglia, whereas white matter and cerebral cortex (as a whole) showed higher CBF2020 (Fig. 2A and Table 1). Comparing TI-dependent CBF values extracted from cortical VOIs revealed that CBF1500 was higher in the insular cortex, whereas CBF2020 was higher in the parietal and occipital cortex (Fig. 2B and Table 1). No significant TI-dependent CBF differences were found in the frontal cortex, temporal cortex, and temporo-mesial VOIs.

**Table 1:** TI-dependent differences of CBF

Brain regions	CBF values [ml/min/100 g]		*p* value
	TI 1500 ms	TI 2020 ms	
Cerebral cortex	38.87 ± 7.05	42.43 ± 9.02	< 0.01
White matter	23.10 ± 4.49	26.79 ± 5.55	< 0.001
Basal ganglia	34.31 ± 6.73	30.09 ± 7.40	< 0.05
Cortical VOIs			
Frontal			
L	37.92 ± 7.67	40.74 ± 9.40	n.s
R	39.26 ± 8.33	41.23 ± 10.34	n.s
Parietal			
L	37.64 ± 7.77	44.72 ± 8.92	< 0.001
R	35.65 ± 6.51	42.93 ± 8.77	< 0.001
Temporal			
L	38.90 ± 8.18	41.30 ± 9.55	n.s
R	40.51 ± 8.39	41.25 ± 9.66	n.s
Occipital			
L	38.43 ± 5.53	47.45 ± 9.19	< 0.01
R	42.40 ± 7.31	50.19 ± 10.49	< 0.01
Insular			
L	43.43 ± 11.00	36.75 ± 10.68	< 0.001
R	41.18 ± 9.53	37.40 ± 11.46	< 0.01
Temporomesial			
L	44.02 ± 10.95	41.37 ± 9.57	n.s
R	44.10 ± 8.84	41.06 ± 8.34	n.s

For each region, the across-subject mean ± standard deviation values are reported [ml/min/100 g], along with the corresponding *p* values reflecting TI-dependent differences—only Wilcoxon significant *p* values surviving Benjamini–Hochberg adjustment are displayed. See Fig. 2 for the corresponding box-plots

*VOIs* volumes of interest, *L* left hemisphere, *R* right hemisphere, *n.s.* non-significant

Furthermore, the voxel-wise CBF subtraction-maps (Fig. 2C) visually showed that:

- *CBF1500 was higher in*: the perisylvian cortex (including insular, temporal, frontal and parietal perisylvian areas), the lateral aspects of the frontal and parietal cortex, the fronto-mesial cortex and the cingulate gyrus, the basal ganglia, some temporo-polar and temporo-mesial areas, some mesial areas at the occipito-temporal junction; voxels clearly corresponding to arterial branches (Willis polygon, middle, anterior, and posterior cerebral arteries);
- *CBF2020 was higher in*: the posterior parietal and occipital cortex, the fronto-polar areas, the centrum semiovale and the external/extreme capsule, the thalamus, the mesencephalon, and the cerebellum.

Overall, CBF1500 was higher in the proximal territories of the arteries (PTAs), and CBF1500 map exhibited higher perfusion signal in the arteries themselves; CBF2020 was higher in the distal territories of the arteries (DTAs), including the so-called ‘watershed areas’, brain areas lying in between distal territories of different arteries (between anterior and middle cerebral artery — ACA-MCA watershed area — or between middle and posterior cerebral artery — MCA-PCA watershed area).

No asymmetry was visually identified in the across-subjects subtraction-maps.

### Differences in *Z*-score of CBF

**Fig. 3:**
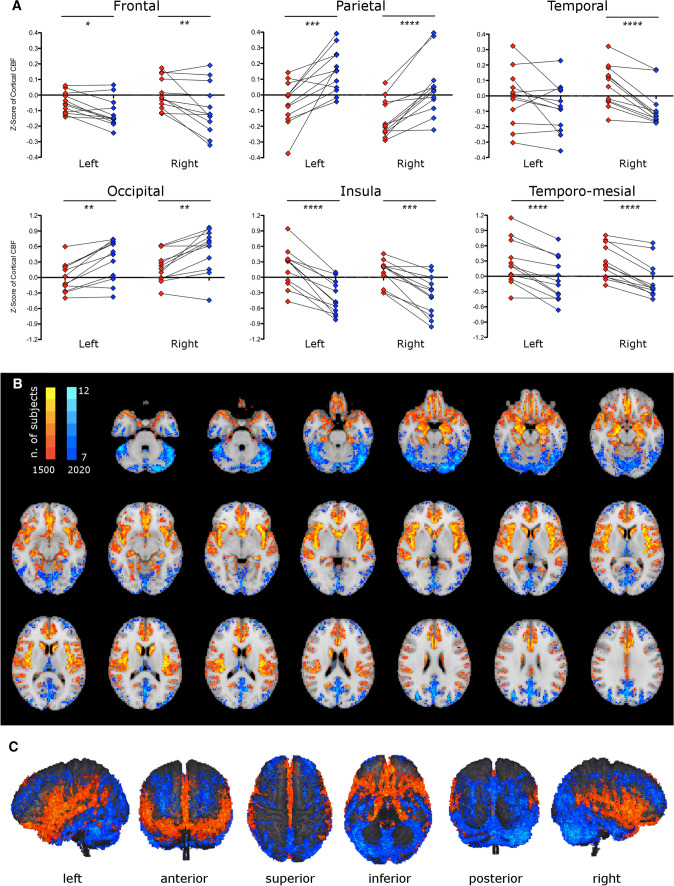
TI-dependent differences of CBF Z-score. Box plots show regional Z-score differences (**A**); *p < 0.05, **p < 0.01, ***p < 0.001, ****p < 0.0001 – only Wilcoxon significant *p* values surviving Benjamini–Hochberg adjustment are displayed. Voxel-wise Z-score across-subjects subtraction-maps (**B**, **C**) illustrate cortical voxels with higher z-1500 (red) and higher z-2020 (blue); color shade is proportional to the number of subjects

Figure 3 summarizes TI-dependent differences of Z-scores both in regional and voxel-wise fashion.

Comparison of VOI Z-scores (Fig. 3A) consistently agreed with the analysis performed on the absolute CBF values, confirming a significant difference in the insular cortex (higher at TI 1500) and in the parietal and occipital cortex (higher at TI 2020). In addition, almost all other cortical VOIs, such as the frontal cortex, temporo-mesial structures, and right temporal cortex showed higher *Z*-scores at TI 1500.

The across-subjects analysis based on the voxel-wise Z-score subtraction-maps (Fig. 3B, C) also provided visually consistent results with those on absolute CBF, revealing a voxel-wise TI-dependent difference in CBF Z-scores approximately in the same grey matter areas (see “Differences in CBF”).

### Scan–rescan analysis

No significant scan–rescan CBF differences were found in any of the VOIs (Suppl. Table 1), with similar subject-specific CBF values in ScanA and ScanB (Suppl. Table 2). The voxel-wise CBF scan–rescan variability was small, with median scan–rescan variations (ScanB–ScanA) ranging from − 1.46 to + 0.31 ml/min/100 g in the grey matter (Suppl. Table 3), corresponding to − 4.6% and + 1.1% respectively. The voxel-wise CBF subtraction-maps (Suppl. Fig. 1) showed that scan–rescan differences were randomly distributed, not corresponding to PTAs/DTAs — unlike the TI-dependent differences in the pediatric patients’ cohort.

### Visual assessment at single-subject level

**Fig. 4:**
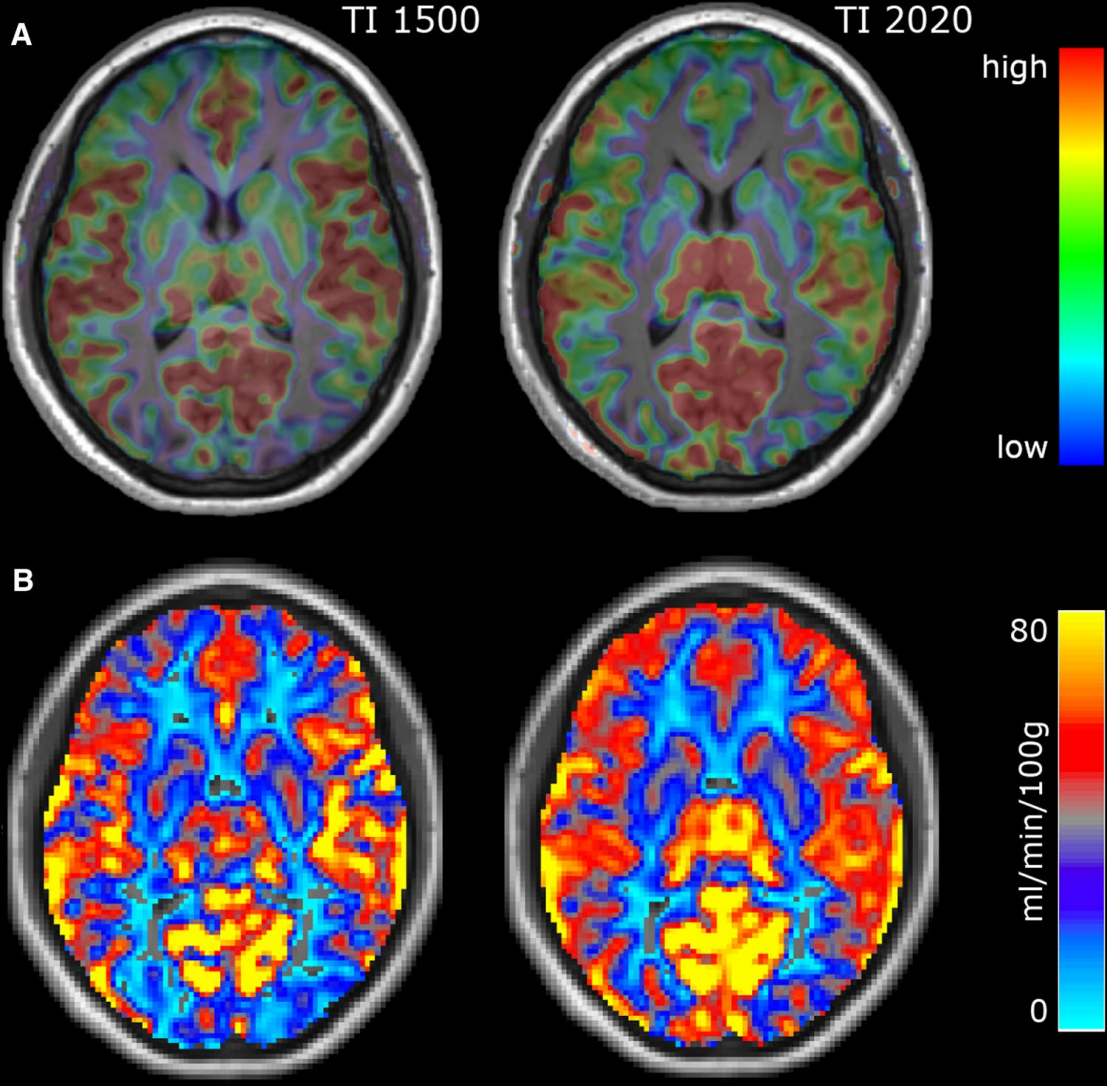
Visual assessment of TI-dependent differences: a representative case. Single-subject perfusion-weighted maps as computed with a clinical software (**A**) and quantitative CBF-maps as computed with FSL (**B**)

As an additional analysis, we explored whether the TI-dependent differences revealed by the across-subject analysis were also evident at the single-subject level. Figure 4 reports images from a representative case from our cohort, including qualitative perfusion-weighted maps as computed with a clinical software (Fig. 4A) and quantitative CBF maps as computed with FSL (Fig. 4B). The TI-dependent perfusion differences were qualitatively evident at single-subject level, both in the qualitative and quantitative maps (see “Visual assessment of perfusion maps” for the computation of the maps). Perfusion signal is higher at TI 1500 in the perisylvian cortex, the fronto-mesial cortex, and the basal ganglia, whereas it is higher at TI 2020 in the posterior occipital and parietal areas (MCA-PCA watershed areas), the thalamus, and the fronto-polar areas (ACA-MCA watershed areas). Furthermore, these maps show that at TI 1500 some watershed areas (MCA-PCA watershed areas, in particular) exhibit a lower perfusion signal than the other cortical areas, mimicking a focal hypoperfused area. Out of 12 patients, the visual inspection of qualitative perfusion-weighted maps and quantitative CBF maps from ASL1500 revealed: in 7 cases a markedly reduced perfusion signal of MCA-PCA and ACA-MCA watersheds compared to the adjacent cerebral regions, in 3 cases a markedly reduced perfusion signal of MCA-PCA watersheds compared to the adjacent areas, in 2 cases a mildly reduced perfusion signal of MCA-PCA watersheds. Conversely, in maps derived from ASL2020, only 3 patients showed a mildly reduced perfusion signal of MCA-PCA watersheds, while 9 out of 12 patients showed no reduction of perfusion signal in the watersheds with respect to the neighbors.

### Impact of sedation and age on TI-dependent changes

**Fig. 5:**
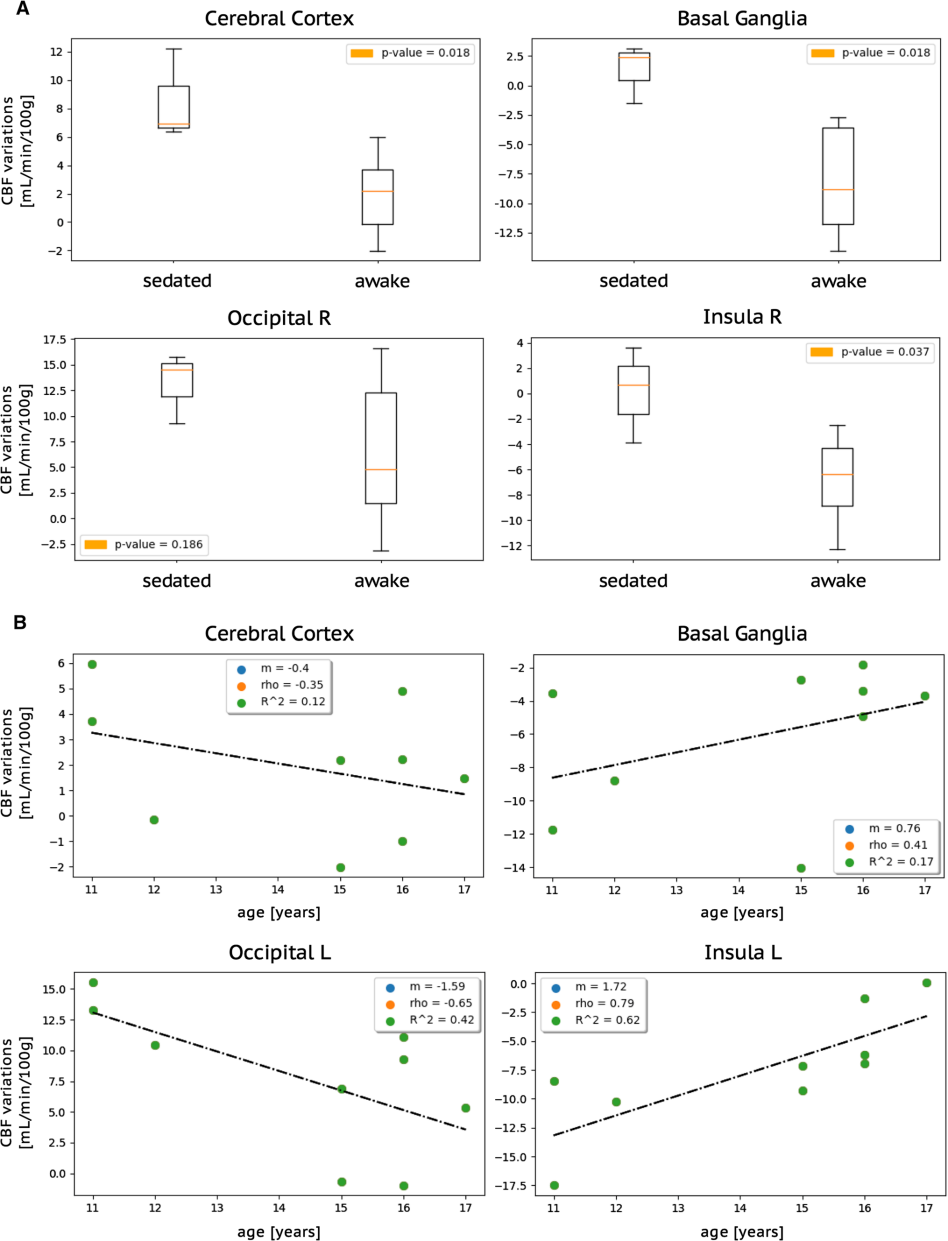
Impact of sedation (**A**) and age (**B**) on TI-dependent changes. Selected VOIs representing DTAs (cerebral cortex as a whole and occipital cortex) and PTAs (basal ganglia and insular cortex) and are displayed. **A** Box-plots displaying median, interquartile range, and range of subject-specific regional CBF-variations (CBF2020–CBF1500) [ml/min/100 g] in sedated and awake patients, along with the corresponding uncorrected Mann–Whitney *p* values – none of which survived a Benjamini–Hochberg adjustment. **B** Scatter-plots of the correlations between age (*x*-axis) and subject-specific regional CBF-variations (*y*-axis), along with correlation coefficients. *L* left hemisphere; *R* right hemisphere; *rho* Pearson correlation coefficient; *m* slope of the regression line; *R*^2^ coefficient of determination

Figure 5 illustrates the impact of sedation (Fig. 5A) and age (Fig. 5B) on TI-dependent changes for selected VOIs representing PTAs (basal ganglia and insular cortex) and DTAs (cerebral cortex as a whole and occipital cortex). Results from the remaining VOIs are reported in Suppl.Fig. 2 (impact of sedation) and Suppl.Tab.4 (impact of age).

*Sedation*: Although no statistical differences survived the Benjamini–Hochberg adjustment, CBF-variations had consistently higher values in sedated patients than in awake patients in all VOIs. That is, in sedated patients TI-dependent changes were more evident in DTAs (where CBF2020 > CBF1500 and CBF-variations > 0 — Fig. 5A, cerebral and occipital cortex) and less evident in PTAs (where CBF1500 > CBF2020 and CBF-variations < 0 — Fig. 5A, basal ganglia and insular cortex). Overall, compared to awake patients, for sedated patients: in DTAs CBF2020 exceeded CBF1500 in a larger amount (e.g. cerebral and occipital cortex); in PTAs either CBF1500 exceeded CBF2020 in a smaller amount (e.g. basal ganglia) or did not exceed CBF2020 (e.g. insular cortex), with some instances of CBF2020 > CBF1500 even in PTAs.

*Age*: CBF-variations and age showed negative correlations in DTAs (Fig. 5B, cerebral and occipital cortex) and positive correlations in PTAs (Fig. 5B, basal ganglia and insular cortex). Throughout VOIs, TI-dependent changes are consistently less evident when age increases, as CBF-variations are closer to zero: they decrease with age (negative correlation) in DTAs (where CBF-variations > 0 since CBF2020 > 1500), and increase (positive correlation) in PTAs (where CBF-variations < 0 since CBF1500 > 2020). That is, in younger patients (aged ~ 11), compared to older ones (aged ~ 17): in DTAs CBF2020 exceeded CBF1500 in a larger amount (e.g. cerebral and occipital cortex); in PTAs CBF1500 exceeded CBF2020 in a larger amount (e.g. basal ganglia and insular cortex).

## Discussion

In this study, we evaluated the impact of the inversion time (TI) on cerebral perfusion estimation by PASL in a pediatric cohort. To our knowledge, this is the first study to provide data regarding variations in perfusion estimation induced by PLD/TI on pediatric subjects.

Our main finding is that there is a region-dependent effect of the choice of TI on CBF, with brain areas adjacent to arterial branches (PTAs) exhibiting higher CBF at TI 1500 ms and distal areas (DTAs), including watershed areas, showing higher CBF at 2020 ms. This result was mostly evident from the voxel-wise across-subjects subtraction-maps. Indeed, the statistical analysis performed on the VOIs returned significant results in cases where the VOI was predominantly composed by either DTAs (i.e. parietal and occipital cortex, showing higher CBF2020) or PTAs (i.e. insular cortex and basal ganglia, showing higher CBF1500). Conversely, the statistical analysis did not provide insightful results for VOIs including both PTAs and DTAs (e.g. frontal and temporal cortex), suggesting that the TI-dependent effect in these two regions is averaged out. In addition, our analyses on CBF *Z*-score demonstrated that TI modification has a similar impact on subject-specific standardized CBF, meaning that TI influences also the relationships among relative perfusion signals of several cortical areas within the same subject, with potential clinical implications when perfusion signals are compared among several brain regions. This was further proven by a visual inspection of both qualitative and quantitative perfusion maps, confirming our results that perfusion signal at TI 1500 ms is higher in the PTAs than in the watershed areas. This visual assessment clearly showed that such TI-dependent changes are also evident from qualitative perfusion evaluations and that are also relevant at the single-subject level. These results were validated by demonstrating a good scan–rescan reproducibility (both with VOI- and voxel-wise approaches) in a cohort of healthy adults.

Overall, our results suggest that PASL at TI 1500 ms, the recommended TI for the pediatric population according to the white paper by Alsop et al. [5], produces CBF maps that may be influenced by the longer ATT in DTAs and in particular in the watershed areas [22]; this can be appreciated both from a quantitative and qualitative evaluation. Conversely, PTAs perfusion in ASL1500 is higher than in ASL2020.

Our results do not allow to define which TI yields the most accurate CBF estimation. However, it is reasonable to make the following considerations. The drawback of a higher TI is a decreased perfusion signal due to an increased labeling decay, while the drawback of a lower TI is obtaining perfusion signal from intraluminal blood that has not reached the capillaries/extracellular space. Therefore, it is possible to speculate that at TI 1500 the higher perfusion in PTAs may be caused by arterial intraluminal labeled water molecules, while the lower perfusion in DTAs may be explained by ATT in DTAs being longer than the selected TI. At TI 2020 DTAs show higher perfusion than at TI 1500, that may potentially correspond to a more accurate estimation, given that a TI increase theoretically should not result in an overestimation (but rather in an underestimation induced by the labeling decay). On the other hand, at TI 2020 PTAs perfusion signal may already be underestimated due to the labeling decay. Overall, our impression is that perfusion in DTAs and in the watersheds may be underestimated at TI 1500. In addition, we speculate that PTA perfusion overestimation at TI 1500 due to intraluminal signal may be more significant than PTA perfusion underestimation at TI 2020 due to the labeling decay, given that at TI 2020 labeling decay does not appear to significantly affect perfusion signal in other regions (e.g. the watersheds). These hypotheses regarding perfusion over/under-estimation at different TIs should be considered as speculations, and should be further assessed in future studies.

Previous studies, mainly on adult volunteers, already addressed ATT effects and intravascular perfusion signal effects, demonstrating that such phenomena may influence CBF estimates in the DTAs and PTAs, respectively [22–30]. A recent study [31] reported comparable results on their cohort of young healthy adults (age range 20–44), as they reported CBF gradually decreasing along with the increase of TI values (1525 ms, 2025 ms, 2525 ms) in VOIs corresponding to PTAs (e.g. basal ganglia, insular cortex, Heschl gyrus, superior temporal gyrus, frontal opercular areas, and cingulate gyrus) and gradually increasing in VOIs corresponding to the watershed areas (e.g. superior frontal regions, inferior temporal, temporal pole, and superior parietal regions). Other studies [32, 33] computed ATT values for pediatric subjects. Jain et al. [32] reported a mean ATT of 1538 ± 123 ms across-subjects (aged 7–17 years), with 14 out of 22 observations exceeding 1500 ms (and up to ~ 1700 ms). Since these values refer to the mean ATT, it can be assumed that ATT in DTAs was significantly higher than 1500 ms for many subjects. Hu et al. [33] reported mean ATT values reaching ~ 1400–1500 ms in some subjects, for which ATT in DTAs may therefore exceed 1500 ms—despite the enrolled subjects being younger (average age 8.6 years). These results further support our hypothesis of DTAs perfusion being underestimated at TI 1500 ms.

Our hypothesis of perfusion being overestimated due to arterial perfusion signal in PTAs is further supported by the findings reported by Ferré et al. [34] on their cohort of healthy adults (age range 21–56) imaged at multiple TIs (ranging 1200–1800 ms), describing arterial artifacts at lower TIs, progressively decreasing along with the increase of TI.

In addition, to some extent potential ATT differences between anterior and posterior circulation could contribute to TI-dependent perfusion differences, indeed hypoperfusion was always more evident in posterior (MCA-PCA) rather than anterior (ACA-MCA) watersheds and the thalamus showed higher perfusion at TI 2020. Previous studies support this finding, as MCA-PCA watersheds were reported to have longer ATT than ACA-MCA watersheds [27] and PCA territories longer than ACA and MCA territories [33].

As additional analyses, we reported on a potential impact of sedation and age on TI-dependent changes.

For sedated patients: in DTAs CBF at 2020 ms is higher than at 1500 ms in a larger amount than in awake patients, while it is comparable to 1500 ms in the PTAs. For younger patients (aged ~ 11): in DTAs CBF at 2020 ms is higher than at 1500 ms in a larger amount than in older patients, and lower than at 1500 ms in a larger amount in the PTAs. Although these results were obtained from small subsets of patients and should be taken cautiously, they suggest that TI should be set even more carefully in younger patients, for which TI-dependent changes were more pronounced in both DTAs and PTAs. We speculate that these results may once again be due to an increased ATT in sedated and younger patients, given that labeling efficiency and decay are less likely to be significantly affected by sedation and age.

If proven, a longer ATT in sedated patients could be potentially ascribable to the vasodilating effects of sevoflurane [35, 36]. In fact, based on the current knowledge regarding the effects of sevoflurane, the increase in the cross-sectional surface of the cerebral [35] and non-cerebral [36] vascular structures (i.e. vasodilation) are not compensated by a cardiac output increase. On the contrary, the myocardial function may be depressed [37]. These phenomena could theoretically result in a lower blood velocity in the arterioles, and therefore a longer ATT.

On the other hand, the hypothesis of a longer ATT in younger patients would be in contrast with a previous study that specifically focused on ATT quantification in a pediatric cohort (aged 7–17) years and reported shorter ATT in younger patients [32].

### Clinical implications

The potential perfusion underestimation in DTAs, and in particular in the watershed areas, at TI 1500 ms may have implications when perfusion-weighted maps are qualitatively evaluated by neuroradiologists in clinical settings, as the low perfusion signal in the watershed areas might be mistaken for focal hypoperfused areas. This may occur during the evaluation of seizure-induced perfusion alterations, as the epileptic focus may be detected as a hypoperfused cortical area on ASL. This potential impact on the clinics is also supported by recent findings on adult patients with Alzheimer’s disease (AD). Kaneta and colleagues [38] reported the ‘border zone sign’, consisting of hypoperfusion of the ACA-MCA and MCA-PCA watershed areas and supposedly correlated to AD, being more likely to be identified at PLD 1500 ms than 2500 ms on PCASL. Nevertheless, at least three other studies [38–41] reported on the possibility of a false-positive ‘border zone sign’ on PCASL with PLD ~ 1500 ms in up to 70% of cases [39]. Indeed, these studies demonstrated an ASL-SPECT or ASL-PET mismatch, as ASL ‘border zone sign’ was not recognized on SPECT or PET images of the same patients in several cases. In the light of our results, we support the hypothesis of false-positive watershed hypoperfusion being due to a short PLD/TI, as already suggested by these authors. Potentially, this effect may be even more problematic in the evaluation of AD patients, as ATT is longer in the elderly population.

### Limitations and future perspectives

This study has some limitations. First, the reduced number of enrolled subjects. Second, imaging data from other perfusion modalities were not available (e.g. SPECT or Dynamic Susceptibility Imaging), preventing a potential comparison aimed at further evaluating whether ASL1500 or ASL2020 should be considered more quantitatively accurate. Third, similarly, the absence in the literature of reliable reference values for normal regional ASL-derived CBF in the pediatric population prevented a definitive evaluation of TI-dependent CBF under/over-estimations. Fourth, the subjects enrolled were affected by pathological conditions; nevertheless, we exclusively performed intra-VOI/intra-voxel comparisons between TIs, to prevent any impact of potential perfusion differences among subjects or between left/right sides caused by the underlying pathological conditions.

Overall, our results seem to suggest that TI 2020 may result in a more accurate estimation of ASL-prefusion in the pediatric population. In fact, setting the TI to 1500 ms, as recommended in the white paper [5], may lead to both PTAs perfusion overestimation due to intra-vascular signal contributions and to DTAs perfusion underestimation due to local ATT being longer than TI. In the light of these findings, future studies should explore the possibility of employing a TI/PLD longer than 1500 ms in order to assess whether the recommended PLD/TI should be revised. Further evidence could be provided by multimodal analyses including PET/SPECT datasets, and from multi-TI ASL with ATT quantifications and improved quantitative perfusion maps [4, 41–44]. In this scenario, additional imaging strategies were proven useful to achieve refined perfusion quantifications, and could serve as reference techniques to validate results from standard ASL. For instance, ASL with crusher gradients eliminates intra-vascular signal contributions [26], and vessel-encoded ASL provides more accurate perfusion estimates in watershed territories [45]. Another promising approach is velocity-selective ASL, in which all spins flowing with a specified velocity are tagged. Since the labeling is not spatially-selective, the CBF quantification provided by this technique is insensitive to ATT-related effects [46]. In a recent study, velocity-selective ASL reliably quantified CBF in Moyamoya pediatric patients, whereas traditional PASL dramatically underestimated CBF in territories with disease-related ATT increase [47].

Finally, validation through correlations with clinical data is required for definitive recommendations, in order to assess how the diagnostic accuracy may be affected by PLD/TI when identifying perfusion alterations in pathological conditions.

## Conclusions

In our pediatric cohort, PASL brain perfusion was affected by the value of TI with a region-dependent effect, with proximal and distal territories of the arteries showing higher perfusion (quantitatively and qualitatively) at 1500 ms and at 2020 ms, respectively. The presence of intraluminal arterial signal in the proximal territories of the arteries and the longer arterial transit time in the distal territories, including the watershed areas, may account for this difference. Additional data are necessary in order to define the optimal TI for this age category and to correlate PASL at different TIs with clinical features of the patients. However, our results suggest that ASL performed with short PLD/TI (1500 ms is the recommended for the pediatric population) may result in a lower perfusion signal of the watershed areas. Clinical neuroradiologists should be aware of this finding, as it could mimic focal hypoperfused areas that may be erroneously interpreted as focal seizure foci in epileptic patients.

## Supplementary Information

Below is the link to the electronic supplementary material.

Supplementary file1 (PDF 736 kb)

## Data Availability

Datasets employed in this study are available from the authors upon request.

## Abbreviations

ACA: Anterior cerebral artery
ASL: Arterial spin labeling
ATT: Arterial transit time
CBF: Cerebral blood flow
DTAs: Distal territories of the arteries
MCA: Middle cerebral artery
PASL: Pulsed ASL
PCASL: Pseudo-continuous ASL
PLD: Post-labeling delay
PCA: Posterior cerebral artery
PTAs: Proximal territories of the arteries
SNR: Signal to noise ratio
TI: Inversion time
VOI: Volume of interest

## Code availability

In-house code employed in this study is available from the authors upon request.
