# Supplementary material for: Impact of the inversion time on regional brain perfusion estimation with clinical arterial spin labeling protocols
Source: MAGMA. 2021 Oct 13;35(3):349–63. doi: 10.1007/s10334-021-00964-7 (PMC9188620; doi:10.1007/s10334-021-00964-7)
Supplement: Supplementary file 1 — Supplementary file1 (PDF 736 kb) [file 10334_2021_964_MOESM1_ESM.pdf]

Supplementary Materials
Impact of the Inversion Time on regional brain perfusion estimation with clinical Arterial Spin Labeling protocols
Magnetic Resonance Materials in Physics, Biology and Medicine

Supplementary Table 1. Scan-rescan CBF differences in healthy adults. For each region, the across-subject mean ± standard deviation values are reported [ml/min/100g], along with the corresponding p-values reflecting scan-rescan differences – all Wilcoxon comparisons returned non-significant p-values.
VOIs = Volumes of Interest; L = Left hemisphere; R = Right hemisphere; n.s.= non-significant.
[a] For these regions, subject-specific values are reported in Supplementary Table 2.

Brain Regions		CBF values [ml/min/100g]		
		Scan A	Scan B	*p-value*
Cerebral Cortex [a]		34.12 ± 4.96	33.52 ± 4.70	n.s.
White Matter [a]		18.81 ± 3.72	18.43 ± 3.41	n.s.
Basal Ganglia [a]		27.65 ± 2.03	27.70 ± 2.05	n.s.
Cortical VOIs				
Frontal	L	33.44 ± 5.68	32.58 ± 5.21	n.s.
	R	33.38 ± 6.10	33.00 ± 5.51	n.s.
Parietal	L	34.77 ± 6.35	34.23 ± 5.90	n.s.
	R	35.30 ± 5.65	35.13 ± 5.31	n.s.
Temporal	L	32.60 ± 3.62	31.57 ± 3.11	n.s.
	R	33.58 ± 4.99	33.15 ± 5.16	n.s.
Occipital	L	36.18 ± 5.74	35.11 ± 5.66	n.s.
	R	39.15 ± 3.19	38.29 ± 3.55	n.s.
Insular	L	34.55 ± 2.97	33.85 ± 3.41	n.s.
	R	34.97 ± 3.09	34.00 ± 3.10	n.s.
Temporo-mesial	L	28.88 ± 3.25	28.71 ± 3.31	n.s.
	R	29.95 ± 4.08	29.93 ± 4.71	n.s.

Supplementary Table 2. Scan-rescan CBF differences in healthy adults. For each region, the mean subject-specific values are reported [ml/min/100g].

Subject	Regional CBF values [ml/min/100g]					
	Cerebral Cortex		White Matter		Basal Ganglia	
	Scan A	Scan B	Scan A	Scan B	Scan A	Scan B
#01	31.63	30.84	19.58	18.99	27.08	26.66
#02	27.89	28.31	12.82	13.30	26.77	27.84
#03	37.01	36.95	20.92	20.60	30.64	30.25
#04	33.32	31.74	18.20	17.15	25.27	24.91
#05	40.77	39.75	22.55	22.11	28.49	28.85

Supplementary Table 3. Scan-rescan voxel-wise variability of grey matter CBF in healthy adults. For each subject, the histogram statistics of grey matter CBF subtraction maps (Scan B – Scan A) are reported [ml/min/100g].
pc = percentile.

Subject	voxel-wise CBF subtraction [ml/min/100g]				
	10th pc	25th pc	median	75th pc	90th pc
#01	-5.41	-3.11	-0.90	1.24	3.11
#02	-3.36	-1.55	0.31	1.88	3.48
#03	-3.85	-1.99	-0.24	1.57	3.16
#04	-4.82	-3.17	-1.46	0.36	2.04
#05	-4.39	-2.60	-0.88	0.87	2.32

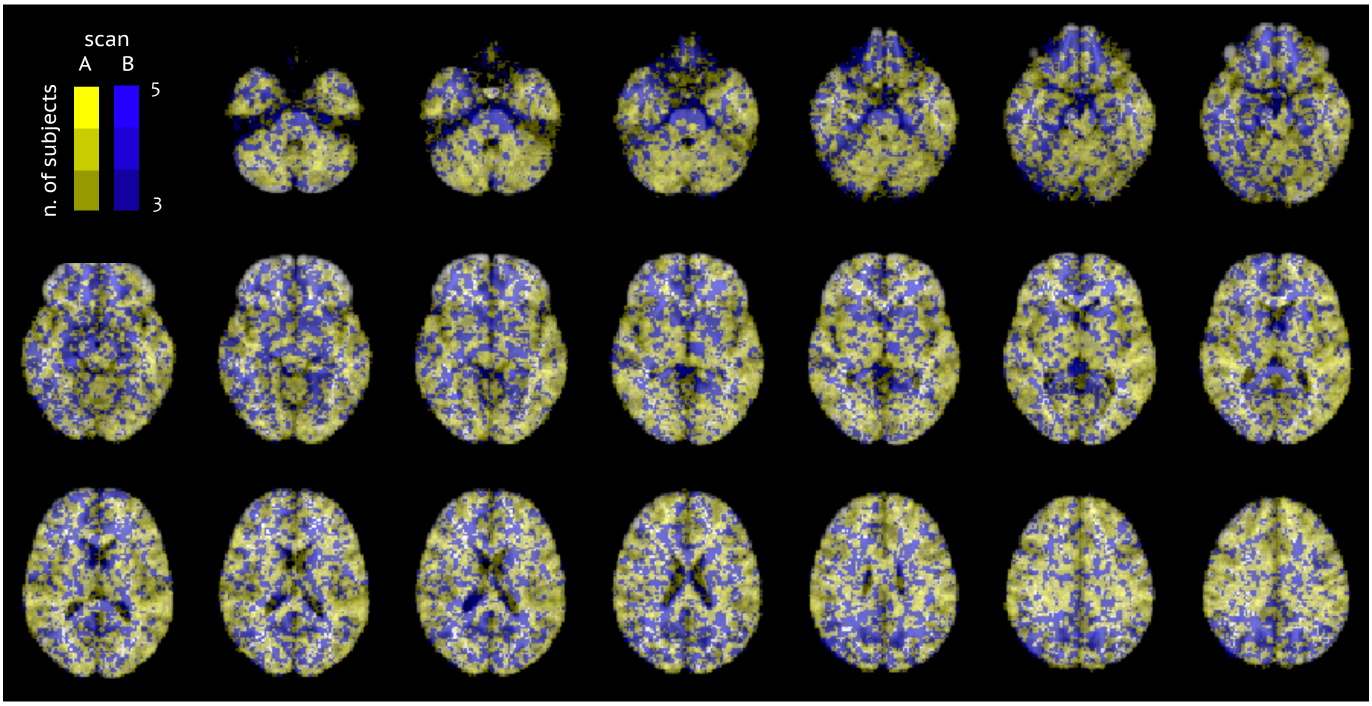

Supplementary Figure 1. Scan-rescan voxel-wise differences of CBF in healthy adults. Voxel-wise CBF across-subjects subtraction-maps illustrate voxels with higher CBF on Scan A (yellow) and higher CBF on Scan B (blue); color shade is proportional to the number of subjects.

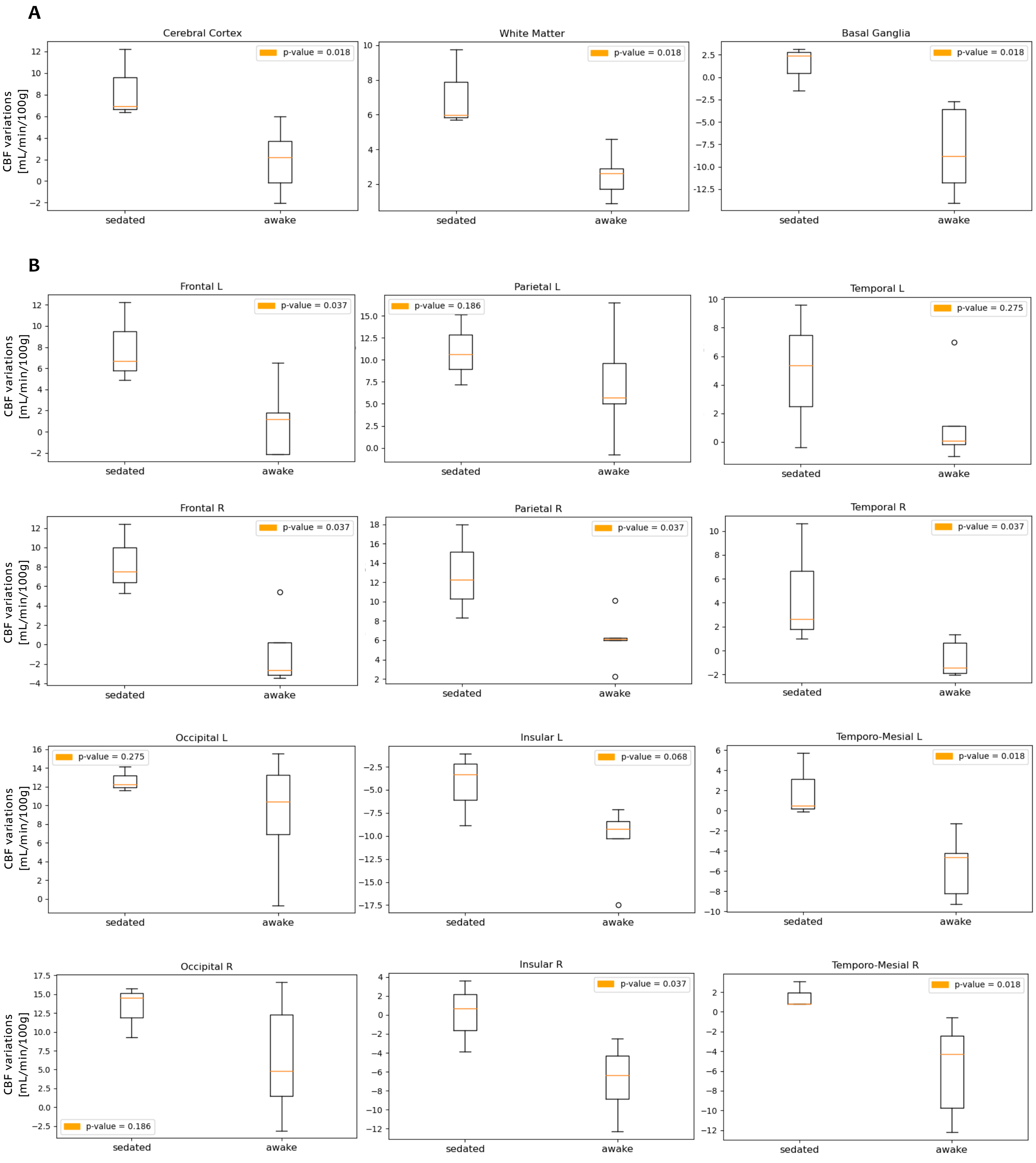

Supplementary Figure 2. Impact of sedation on TI-dependent changes (n=8) in pediatric patients. The subject-specific regional CBF-variations (CBF2020–CBF1500) are displayed as median, interquartile range (box), and range (whiskers) [ml/min/100g]. The corresponding p-values reflecting the differences between 3 sedated and 5 awake pediatric patients are also reported – although none of the Mann-Whitney U-test p-values reported survived a Benjamini-Hochberg adjustment.
L = Left hemisphere; R = Right hemisphere.

Supplementary Table 4. Impact of age on TI-dependent changes (n=9) in pediatric patients. The relationships between age and regional CBF-variations (CBF2020–CBF1500) are reported. The sign of the CBF-variations reflects whether CBF2020 was higher or lower than CBF1500 in a specific region: '+' = all patients had CBF2020>CBF1500; '–' = all patients had CBF1500>CBF2020; 'mainly' refers to the majority of the patients; '+ / – ' = some patients had CBF2020>CBF1500 and some CBF1500>CBF2020. The 'interpretation' column states, in addition to the strength of the correlation, whether the TI-dependent effects changed when age increased: 'less relevant' means that the values of CBF-variations approached zero when age increased, corresponding to either a positive correlation in the presence of negative values of CBF-variations, or negative correlation in the presence of positive values of CBF-variations.
rho = Pearson correlation coefficient; m = slope of the regression line; R^2 = coefficient of determination; VOIs = Volumes of Interest; L = Left hemisphere; R = Right hemisphere; n.s.= non-significant.

Brain Regions		rho	m	R^2	Sign of CBF-variations (CBF2020–CBF1500)	Interpretation: how TI-dependent effects change with age?
Cerebral Cortex		-0.35	-0.4	0.12	mainly + (2020>1500)	less relevant (weak correlation)
White Matter		-0.29	-0.19	0.08	+ (2020>1500)	less relevant (weak correlation)
Basal Ganglia		+0.41	+0.76	0.17	– (1500>2020)	less relevant (moderate correlation)
Cortical VOIs						
Frontal	L	-0.29	-0.34	0.08	mainly + (2020>1500)	less relevant (weak correlation)
	R	+0.02	+0.02	0.00	+ / –	no correlation
Parietal	L	-0.62	-1.49	0.39	mainly + (2020>1500)	less relevant (strong correlation)
	R	-0.47	-0.62	0.22	+ (2020>1500)	less relevant (moderate correlation)
Temporal	L	-0.19	-0.21	0.04	mainly + (2020>1500)	less relevant (weak correlation)
	R	-0.01	-0.01	0.00	+ / –	no correlation
Occipital	L	-0.65	-1.59	0.42	mainly + (2020>1500)	less relevant (strong correlation)
	R	-0.46	-1.35	0.21	mainly + (2020>1500)	less relevant (moderate correlation)
Insular	L	+0.79	+1.72	0.62	– (1500>2020)	less relevant (strong correlation)
	R	+0.72	+1.08	0.52	– (1500>2020)	less relevant (strong correlation)
Temporo-mesial	L	+0.37	+0.37	0.14	mainly – (1500>2020)	less relevant (weak correlation)
	R	+0.21	+0.35	0.04	– (1500>2020)	less relevant (weak correlation)
